# Effect of Hydroxyapatite on the Migration of Fe(III) Ions: Kinetic, Equilibrium and Thermodynamic Study

**DOI:** 10.3390/ma15165652

**Published:** 2022-08-17

**Authors:** Qing Ye, Gongming Qian, Lulu Liu, Fu Yang, Wei Liu

**Affiliations:** 1 College of Resource and Environmental Engineering, Wuhan University of Science & Technology, Wuhan 430081, China; 2 Hubei Key Laboratory for Efficient Utilization and Agglomeration of Metallurgic Mineral Resources, Wuhan University of Science & Technology, Wuhan 430081, China; 3 College of Optical, Mechanical and Electrical Engineering, Zhejiang A & F University, Hangzhou 311300, China

**Keywords:** iron migration, hydroxyapatite, adsorption isotherm, thermodynamic

## Abstract

The recycling and regeneration of Fe(III) is the key point for promoting the oxidation reaction of ore to produce acidic mine drainage (AMD). Hydroxyapatite (HAP) has excellent adsorption ability of Fe(III), which has good biocompatibility and is widely distributed in nature. In order to achieve the source treatment of AMD, the migration and transformation of Fe(III) in the presence of HAP were systematically investigated. In this study, the influence of HAP on the migration of Fe(III) was evaluated though the transformation capacity of Fe(III) by HAP. The adsorption transformation kinetic, equilibrium and thermodynamics of Fe(III) using HAP were also systematic investigated. The transformation efficiency of Fe(III) increased with the increasing initial pH value and reached 99.8% at a pH of 5 due to the hydrolysis reaction. The transformation efficiency was also up to 99% at an initial pH of 2 when the reaction temperature increased to 313 K. However, the transformation capability of Fe(III) decreases with reaction temperature. The kinetics of the adsorption of Fe(III) fitted the pseudo second order kinetic model. Experimental results were also analyzed by the Langmuir isotherm equations at room temperatures. R_L_ separation factor for Langmuir isotherm showed that the migration of Fe(III) is successfully hindered by HAP. Various thermodynamic parameters such as enthalpy (ΔH), Gibbs free energy (ΔG) and entropy (ΔS) changes were computed, which showed that the transport lag of Fe(III) caused by HAP is spontaneous and endothermic.

## 1. Introduction

Due to the exposure of sulfur minerals on the Earth’s surface made by the mining, quarrying and civil construction, it can react with certain bacteria, oxygen and water, generating sulfuric acid, bivalent and trivalent iron ions and other metal ions to form acidic mine drainage (AMD) [1,2,3]. Because of its high acidity and high ion concentration, AMD has a great impact on the ecological environment [4]. Pyrite is an abundant sulfide mineral on the planet and its oxidation process is shown in Equations (1)–(3) [5,6]. Pyrite oxidation is a multistep process and the regeneration of ferric iron (Equations (2) and (3)) is the key reaction in the promoting oxidation. So far, the main treatment methods of AMD can be divided into the prevention and control of AMD in terms of its origins, deployment of acid drainage migration prevention measures and the collection and treatment of effluent [7,8]. Due to the high cost of sewage treatment, the treatment methods of AMD are increasingly inclined towards source control [9], such as covers [10], alkaline addition [11], bacterial inhibition and passivation [12]. The essence of these AMD treatment methods is to hinder the release and the migration process of iron. So, exploring the mechanism of iron migration in AMD is essential to achieving AMD treatment at its origins.

(1) $$2{\mathrm{FeS}}_{2}+7O_{2}+2H_{2}O=2{\mathrm{Fe}}^{2+}+4{\mathrm{SO}}_{4}^{2-}+4H^{+}$$

(2) $$4{\mathrm{Fe}}^{2+}+O_{2}+4H^{+}=4{\mathrm{Fe}}^{3+}+2H_{2}O$$

(3) $$2{\mathrm{FeS}}_{2}+14{\mathrm{Fe}}^{3+}+4H_{2}O=15{\mathrm{Fe}}^{2+}+2{\mathrm{SO}}_{4}^{2-}+16H^{+}.$$

Hydroxyapatite (HAP) is the main component of bone and has good biocompatibility, which can be widely used in the medical and environmental protection industries [13,14]. Due to the surface properties and internal channels structure of HAP, Ca^2+^ and OH^−^ are easily exchanged by ions similar to lattice ions respectively [15,16]. Therefore, HAP can be regarded as an ideal adsorptive material and a superior covers material for long-term containments because of its high sorption capacity for heavy metals, low water solubility, high stability under redox conditions, availability and low cost. The relevant literature reported the use of HAP for the removal of Fe(II) in batch experiments [17]. In removal experiments, only some retention of Fe(II) on new phases but no Fe^2+^ solids were detected by XRD. The interaction of different phosphate species contributed to the in-situ subsurface co-precipitation mechanism of phosphate-Fe(III) [18]. In addition, the apatiteII^TM^ could react with acid water, releasing phosphate and increasing pH value, which induced metal ions to precipitate as metal-phosphates [19]. The experimental results indicated that the iron removal efficiency and the maximum capacity reached 98% and 124.2 mg/g, respectively, at the initial pH value of 4. In a previous study, Fe(III) removal experiments, using natural apatite [20] and HAP [21], were systematically investigated. However, the migration and transformation mechanisms of Fe(III) from aqueous solutions were not clear. The main factor and control link of Fe(III) transformation also need to be further investigated [22].

Above all, in terms of the process of the release, migration and transformation of iron in AMD, HAP can hinder the migration of iron along with AMD flow, reduce the migration ability of iron, increase the iron migration lag coefficient and prevent iron from spreading around and causing pollution. On the other hand, HAP performs weakly and is alkaline after dissolving and can neutralize acidity, which is feasible for controlling apatite treatment of AMD at the source control. Thus, it is very important to investigate the mechanism of iron migration in AMD with the presence of HAP, and it will guide the research on iron’s biogeochemical behavior in AMD.

In order to prevent the influence of the oxidation reaction of acid radical anions, the Fe(NO_3_)_3_ solution was used to investigated the migration behavior of iron ions. HAP used in this study was prepared by natural apatite, which is the kind of siliceous phosphate rock containing about 20% P_2_O_5_, quartz, dolomite and limestone [23]. The influencing factors, including the pH of the solution, the initial Fe(III) concentration, reaction time, dosage of the HAP and reaction temperature, were systematically investigated. In addition, the migration mechanism of Fe(III) and the main control step in the transformation process in AMD with the presence of hydroxyapatite were discussed.

## 2. Materials and Methods

### 2.1. Materials

The purity of ammonia hydroxide [NH_4_OH] used for the hydroxyapatite synthesized process was above 28%. The Fe(NO_3_)_3_ used to prepare the iron-bearing solution was analytically pure and the brand was Aladdin.

### 2.2. Methods

Hydroxyapatite was synthesized using the wet chemistry method, which is a relatively simple solution process using solution dissolved by natural apatite and stronger ammonia hydroxide [NH_4_OH] as raw materials. In the typical synthesis of HAP nanoparticles, solution dissolved by natural apatite was added drop wisely into solution with ammonia at ambient conditions for 30 min. The reaction pH was controlled by the ammonia at around 10–11. After being aged for 24 h, the reacted production was filtered to separate solid from liquid phase. Then the solid was washed with deionized water until the pH is 7 and dried at 105 °C overnight. The dried mass was heated at 750 °C for 2 h.

HAP and 100 mL of Fe(III) solutions having different concentrations (5, 10, 20, 50, 100, 150 and 200 mg/L) and the pH (1–7) of iron solutions were adjusted with 0.1 M HNO_3_ and 0.1 M NH_3_·H_2_O. In order to investigate the effect of the temperature on the transfer of Fe(III), and several reaction temperatures (273, 283, 298, 313, and 333 K) were systemically studied. The suspensions were stirred with a magnetic stirring bar inside the reactor for different durations. Then, the suspensions were filtered through a 0.22 µm membrane filter and the Fe(III) concentration in the filtrate was analyzed using UV/Vis spectrophotometer (Shimadzu, UV-2550, Kyoto, Japan) at 510 nm [24]. The crystal morphology and structure of HAP before and after reaction were observed using a scanning electron microscope (SEM), an energy dispersive spectrometer (EDS), X-ray Diffraction (XRD) and Fourier transform infrared spectroscopy (FTIR).

## 3. Result and Discussion 

### 3.1. Characterization of Hydroxyapatite

**Figure 1:**
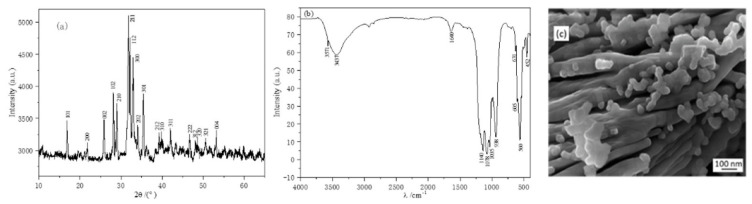
The characterization of HAP: (**a**) XRD; (**b**) FTIR; (**c**) SEM.

The micromorphology characterization of the synthesized crystal particle is presented in Figure 1. The XRD analysis shows that the diffraction peaks of HAP (Figure 1a) agree well with JCPDS card No. 09-0432. The interplanar spacing of the synthesized crystal particle can be calculated based on the data in Figure 1 by the Scherrer formula, as shown in Equation (4):

d = kλ/(Bcosθ),(4)

where λ is the wavelength, B is the full width at half maximum, θ is the diffraction angle and k is the Scherrer constant 0.89. 

According to the highest characteristic peak for HAP {211} at 2θ = 31^◦^, the calculated interplanar spacing is 14.6 nm. Figure 1b shows the FTIR spectra of HAP in the range of 4000–400 cm^−1^. Bands related to the water molecules of hydration at about 1640 cm^−1^ (δOH of hydration water molecules) and at approximately 3437 cm^−1^ (mOH of hydration water molecules) were also present. The recorded symmetric P–O stretching vibration of the PO_4_^3−^ band at 938 cm^−1^, the triply degenerated bending vibrations at 605 cm^−1^, as well as the band at 452 cm^−1^, are attributed to the HAP [25]. The adsorption bands at 1140 cm^−1^ can be assigned to the P–O–H deformation modes, which indicate the presence of the HPO_4_^2−^ ionic group in calcium deficient HAP. The characteristic adsorption bands at 3571 and 631 cm^−1^ that correspond, respectively, to the stretching vibration and bending deformation modes of the lattice OH^−^ ions, indicate the formation of the crystalline HAP phase. The characteristic bands for PO_4_^3−^ also appear at 1078, 1035, 605, 560 and 452 cm^−1^. The SEM micrograph of HAP consisted of a lamellar structure and some agglomerated particles.

### 3.2. Transformation and Migration Behavior of Fe(III)

#### 3.2.1. Effect of Initial pH

**Figure 2:**
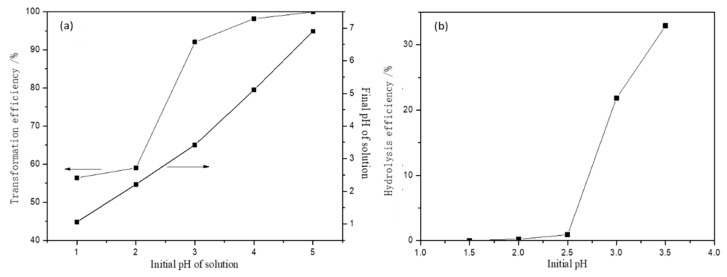
Effect of initial pH on the Fe(III) transformation efficiency in the presence of HAP (**a**) and on the hydrolysis efficiency of Fe(III) in the solution (**b**).

One of the most critical parameters in the migration and transformation process of metal ions is the pH of the medium. Hence, the effect of initial pH on the migration of Fe(III) by HAP was studied first. The initial pH values ranged from 1 to 7 at room temperature (20 °C) and the initial concentration of Fe(III) was 100 mg/L with a HAP dosage of 1 g/L for the reaction time of 30 min. The variation of the transformation efficiency of Fe(III) and the final pH value of the solution are shown in Figure 2a. It can be observed that the transformation efficiency increased dramatically with the increasing initial pH value, and reached 99.98% when the solution’s initial pH value is 5. The change of the final pH value of the solution had a similar trend and also increased with increasing the initial pH; the stepwise transformation of Fe(III) leads to the change of the pH value of the solution in the presence of HAP. It is known that metal ions convert to insoluble hydroxide and precipitated from the solution at higher values, which led to the migration of metal ions of the solution. Therefore, both adsorption and precipitation may exist in the migration and transformation of Fe(III) in AMD.

To analyze the migration and transformation mechanisms of Fe(III), the effect of pH on the hydrolysis of Fe(III) was investigated. The initial concentration of Fe(III) was 50 mg/L whereas the initial pH values were adjusted to 1.5, 2.0, 2.5, 3.0, 3.5 by HNO_3_ and NH_3_·H_2_O solutions. The solutions were filtered after stirring for 10 min and the Fe(III) concentration in the filtrate was analyzed. The hydrolysis efficiency was calculated, as shown in Equation (5):

(5) $$\alpha =\left(C_{0}-C_{t}\right)/C_{0},$$

where α is hydrolysis efficiency, *C*_0_ and *C_t_* are the initial concentration and the filter concentration of Fe(III), respectively.

The result (Figure 2b) indicated that the hydrolysis of Fe(III) in the solution of pH < 2.5 was not strong. When the pH value was beyond 2.5, the hydrolysis efficiency increased dramatically with increasing pH value. This means that the insoluble iron hydroxide also increased and the hydrolysis of Fe(III) may be easier to follow with a higher pH value. As shown in Figure 2b, the transformation efficiency of Fe was 1.40% for the hydrolysis of Fe(III) when the solution’s initial pH was 2 (see Figure 2), the transformation efficiency of Fe using HAP as treating agent can reach 58.63% under the same condition. Hence, the adsorption of HAP is the main reason for the transformation of Fe(III) in an acid environment. Because the pH value of AMD is usually lower than 2, the adsorption of HAP is the main reason that HAP hinders the migration of Fe(III) along with AMD flow. 

**Figure 3:**
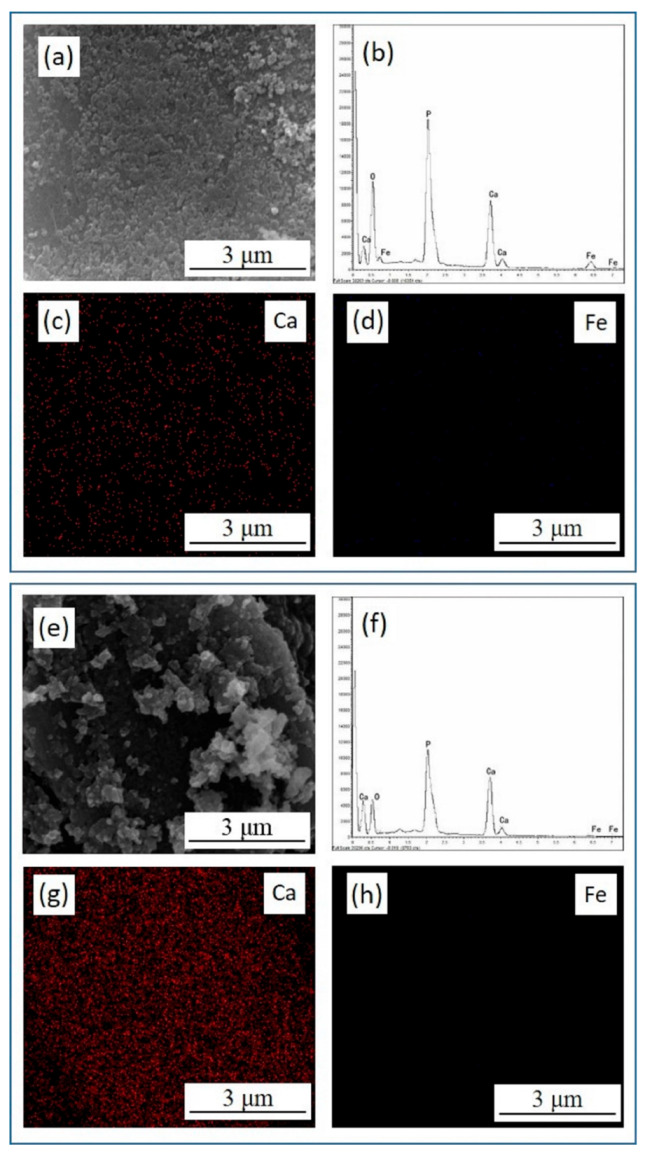
The EDS energy spectrum diagram, the SEM micrographs and the element distribution of the reaction solid products of Fe(III) transformation in presence of HAP at initial pH 2 (**a**–**d**) and pH 5 (**e**–**h**).

In order to investigate the migration and transformation behavior of Fe(III) in the presence of HAP at different initial pHs, the reaction solid products were characterized by SEM-EDS analyses, as shown in Table 1 and Figure 3. The reaction solid production at an initial pH of 2 had an obvious Fe peak and the Fe element was uniformly distributed on the surface of HAP (Figure 3b,d). The atomic ratio of Ca to P (Ca/P) and the transformation efficiency of Fe using HAP were calculated. As shown in Table 1, the Ca/P on the surface of HAP was 1.29, which was less than the standard specific value of 1.67, indicating that there are many metal adsorption sites on the surface of HAP for adsorbing Fe(III). When the initial solution pH was 2, the Ca/P on the surface of reaction solid products was 0.70, which was much less than that of pure synthesis HAP. While the Ca/P on the surface of reaction solid production increased to 1.17 when the solution’s initial pH was 5. The Fe atomic content also first increased when the initial pH value was 2, which illustrated that the ion exchange between Fe(III) and Ca was generated and then absorbed on the surface of HAP at a low initial pH. Combined with the analysis shown in Figure 2a, the transformation efficiency of Fe(III) reached 99.8% at an initial pH of 5, while Fe atomic content decreased obviously. This phenomenon indicated that the Fe(III) hydrolysis was the main migration mechanism, and agreed well with the analysis of Figure 2b.

**Figure 4:**
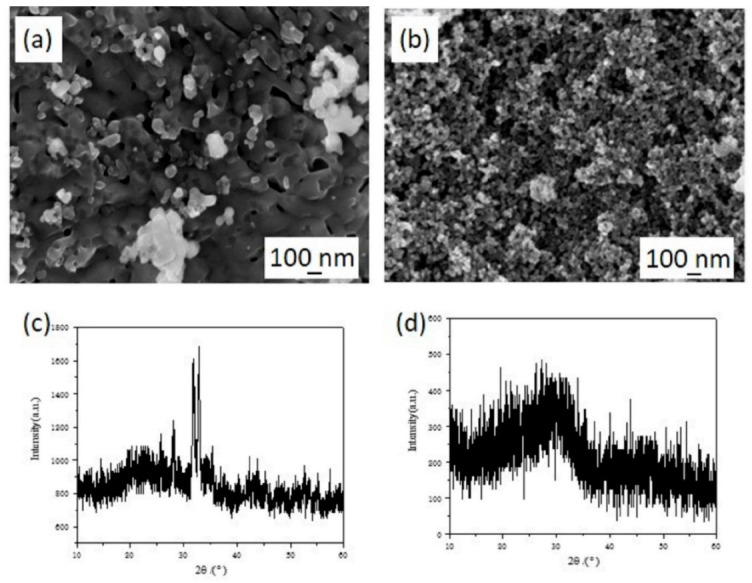
The SEM micrographs and XRD patterns of the reaction solid production of Fe(III) transformation in presence of HAP at reaction time *t* = 10 min (**a**,**c**) and *t* = 2 h (**b**,**d**).

The solubility of HAP in an acid environment may influence the migration of Fe(III) [26]. The micromorphology and phase composition of Fe(III) transformation products by HAP (initial pH = 2.0) are shown in Figure 4. Compared with synthesized HAP (Figure 1c), the surface of HAP became more smooth and the particle size decreased (Figure 4a,b) in the solution with a pH of 2, which revealed that the surface of HAP would dissolve in a solution at a low pH value. As shown in Figure 4c,d, the characteristic peak in XRD patterns of the reaction solid products disappeared after reaction and the crystal structure of HAP was destroyed. Actually, the dissolution of HAP is positive for the migration of Fe(III) from an aqueous solution. After the dissolution of Ca^2+^, more adsorption sites were exposed on the HAP surface, which became negatively charged and tended to absorb Fe(III) ions, due to the incline of the P-O band while Ca^2+^ escaped from the crystal surface. In addition, the dissolved PO_4_^3−^ could react with Fe(III) to produce FePO_4_ which had poor solubility [11]. The H^+^ ions in the solution can also react with HAP to increase the pH value, which leads to the facile hydrolysis of Fe(III). Above all, the effect of initial pH value on the migration mechanism of Fe(III) could be divided into several parts. At low pH values, the HAP dissolves to produce calcium ions, which were exchanged with Fe(III) and adsorbed on its surface to realize the directional migration of Fe(III) from the solution to the HAP surface. As the pH value increases, the dissolution of HAP was inhibited and the adsorption sites’ metal ions are reduced. The hydrolysis and precipitation of Fe(III) play important roles in the transformation process.

#### 3.2.2. The Effect of Initial Fe(III) Concentration

**Figure 5:**
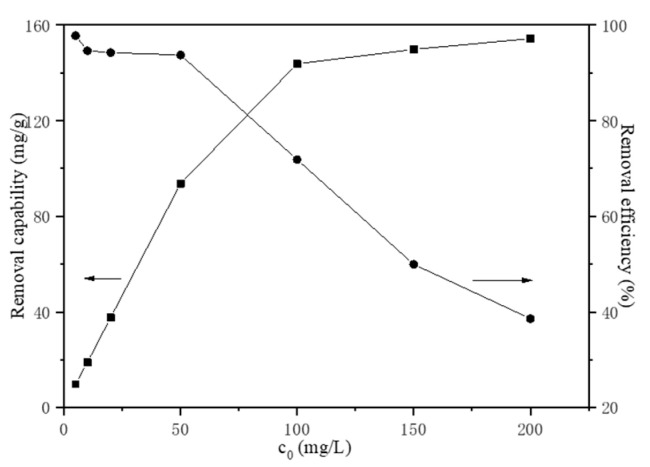
The effect of initial Fe(III) concentration.

The initial Fe(III) concentration has an important effect on the migration mechanism and the effect of initial Fe(III) concentration in the range of 5 to 200 mg/L on adsorption was investigated. The initial pH value was chosen to be pH = 2.0 to avoid hydrolysis of Fe(III) at room temperature (20 °C) with a HAP dosage of 1 g/L for a reaction time of 30 min. As shown in Figure 5, the transformation efficiency of Fe(III) decreased with the increasing initial Fe(III) concentration, while the Fe(III) transformation capability of HAP increased. Generally, the initial Fe(III) concentration provides the necessary driving force to overcome the resistances to the mass transfer of iron between the aqueous phase and the solid phase. The increase in initial Fe(III) concentration also enhances the interaction between iron and apatite powder. Therefore, the increasing of initial Fe(III) concentration enhances the migration of Fe(III), due to the increasing of the concentration gradient driving force. The transformation efficiency of Fe(III) reached 97.80% in the initial concentration of 5 mg/L and decreased to 38.59% in 200 mg/L Fe(III) solution. The experimentally derived maximum transformation capability of HAP was 154.34 mg/g.

#### 3.2.3. The Effect of Reaction Time

**Figure 6:**
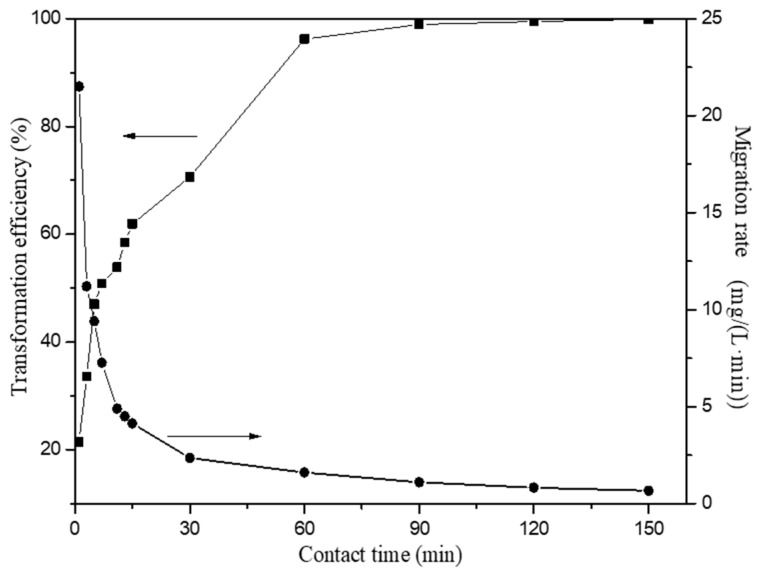
Effect of reaction time on the Fe(III) transformation in the presence of HAP.

The influence of reaction time was investigated in the range of 1 min to 150 min with a HAP dosage of 1 g/L in the initial Fe(III) concentration of 100 mg/L at room temperature (20 °C). The transformation efficiency of Fe(III) using HAP when the initial pH value of 2 is shown in Figure 6. Compared with previous studies, the transformation efficiency of Fe(III) reached above 99% in a shorter reaction time. The transformation efficiency of Fe(III) was high at the beginning due to the large number and availability of HAP surface active sites. The high driving force for the mass transfer caused the rapid migration of Fe(III) at the beginning. The addition of HAP also increases the pH of the solution, which leads to the facile hydrolysis of Fe(III). With the depletion of surface adsorption sites, the migration rate was controlled by the rate at which the adsorbate is migrated from the exterior to the interior sites of HAP particles. With increasing reaction time, the Fe(III) transformation efficiency also increased. After equilibrium within 150 min, the residual Fe(III) is only 0.13 mg/L, which met the requirement of Chinese National Standards of GB21900-2008 (≤3 mg/L).

#### 3.2.4. The Effect of Temperature 

**Figure 7:**
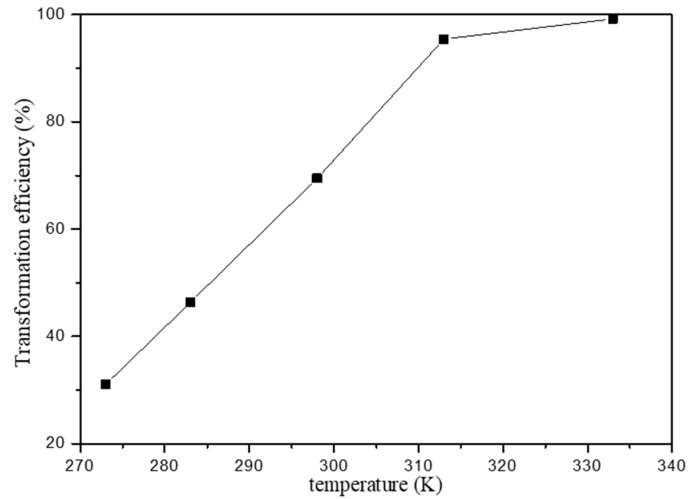
Effect of temperature on the transformation efficiency of Fe(III).

The effect of temperature has a major influence in the sorption process and the temperature effect on Fe(III) transformation was studied in the initial Fe(III) concentration 100 mg/L at pH 2.0. Figure 7 shows the relationship between the transformation efficiency of Fe(III) and temperature. It is found that the Fe(III) transformation increased with increasing temperature. Generally, the adsorption of Fe(III) onto HAP is endothermic, which led to the decreasing of Fe(III)transport ability with the increasing temperature. 

### 3.3. Transformation Mechanisms of Fe(III)

**Figure 8:**
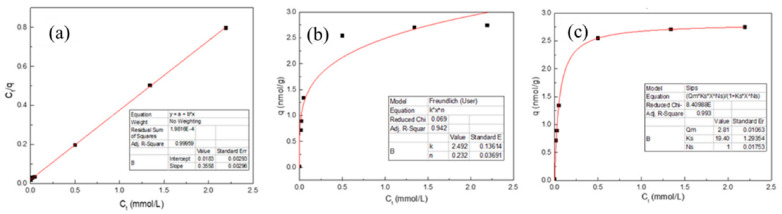
Isotherm adsorption fitting model of Fe(III) in presence of HAP (**a**) Langmuir (**b**), Freundlich and (**c**) Sips.

In order to investigate the adsorption properties of Fe(III) in the migration and transformation process, the experimental equilibrium data were fitted using Langmuir, Freundlich and Sips models (Table 2). Adsorption parameters are as follows: *C_t_* (mmol/L) is the equilibrium concentration of Fe(III) in the solution, q (mmol/g) is the equilibrium concentration of Fe(III) at natural apatite surface, *q_m_* (mmol/g) is the maximum adsorption capacity, K_L_ (L/mmol) the Langmuir constant related to the energy of adsorption and K (mmol^1−n^·L^n^·g^−1^) and n are the Freundlich constants related to the capacity and intensity of the sorption process. Ks is the fitting constants of the Sips model. The graphical illustration of the linear data fitting is shown in Figure 8, while the calculated sorption parameters and the degree of correlation between the sorption data and applied models are listed in Table 2.

It is obvious from Table 2 that the highest correlation coefficient was obtained using the Langmuir model, suggesting that the adsorption of Fe(III) by HAP was monolayer adsorption. Furthermore, the *q_m_* value calculated by this equation corresponded well with the maximal adsorption capacity obtained in the experiment which indicates that the Langmuir equation better fits the experimental data.

The feasibility of the isotherm can be tested by calculating the dimensionless constant separation factor or equilibrium parameter, R_L_ [27]. For initial Fe(III) concentration in the range of 5 to 200 mg/L, used in this study, calculated R_L_ values were between 0.366 and 0.014, indicating that it was conducive to the adsorption of Fe(III) in the presence of HAP. The adsorption process was irreversible, preventing the recycling and regeneration of Fe(III) ions. 

Thermodynamic parameters, such as standard Gibbs free energy change (ΔGº), enthalpy change (ΔHº) and entropy change (ΔSº) at equilibrium at different temperatures, can be calculated from the constant (b, L/mol) of Langmuir isotherm equation as the following equations [28]:

ΔG^o^ = −RT lnb. (6)

In order to use b in the thermodynamic calculations, the value of b expressed in L/mmol in the Langmuir isotherm equation can be multiplied by 1000 to convert the units in L/mol, and ΔHº and ΔSº were obtained from Equation (7).

(7) $$\ln b=\frac{{\Delta S}^{0}}{R}-\frac{{\Delta H}^{0}}{\mathrm{RT}}.$$

The thermodynamic parameters viz. standard free energy change (ΔG^0^), standard enthalpy change (ΔH^0^) and standard entropy change (ΔS^0^) at different temperatures of 293 K, 313 K and 333 K on the optimized condition were calculated and are presented in Table 3. The negative values of ΔG^0^ indicated the spontaneity of the sorption reaction. The positive values of ΔH^0^ indicated the endothermic nature of the sorption process. The positive value of ΔS^0^ showed the increasing randomness at the solid/liquid interface during sorption of Fe(III).

In order to design a fast and effective model, adsorption rate was investigated. A suitable model is required to understand the mechanism of adsorption, such as a mass transfer chemical reaction. The kinetic models viz. pseudo first order, pseudo second order, and intraparticle diffusion models were applied to test the fitness of experimental data. The slope of the plot log(*q_m_* − *q*) vs. *t* of a pseudo-first-order equation [29] would give the values of the rate constant, as shown in Table 4. The pseudo-second-order plot of *t*/*q_m_* vs. *t* [30] resulted in a straight line with higher R^2^ values than the pseudo first order, which indicated the better applicability of the pseudo second order model.

The adsorbate species are most probably migrated from the bulk of the solution into the solid phase through an intraparticle diffusion process, which is often the rate-limiting step in many adsorption processes. The intraparticle diffusion models were applied and the values of rate constants *k_p_*, *C* and the corresponding linear regression correlation coefficient square R^2^ are presented in Table 4.

## 4. Conclusions and Future Perspectives

The present investigation showed that HAP clearly has an influence on the migration of Fe(III) from AMD, and the migration of Fe(III) can be successfully hindered by HAP. The migration of Fe(III) in the presence of HAP was found to be dependent upon pH, reaction time, initial concentration, dosage of the adsorbent and temperature. The transformation efficiency of Fe(III) increases with the increase of HAP dosage and decreases with the increase of initial Fe(III) concentration. The transformation efficiency of Fe(III) in the presence of HAP reached 99 % with a HAP dosage of 1 g/L in the initial Fe(III) concentration of 100 mg/L at room temperature (20 °C) at an initial pH value of 2.0 for 60 min. In addition, the transport ability of Fe(III) decreases with the increase of apatite dosage and increases with the increase of initial iron concentration. The equilibrium data were analyzed using Langmuir, Sips and Freundlich isotherms. Based on the correlation coefficient values, the experimental data yielded excellent fits within the following isotherms order: Langmuir > Sips > Freundlich. It showed that the adsorption process conformed to the Langmuir equation, indicating that the Fe(III) adsorption in the presence of HAP was monolayer adsorption. The dynamic results further clarified that the adsorption on the surface of HAP conformed to the pseudo second order kinetic equation and belonged to chemical adsorption. The initial stage of the adsorption reaction agreed well with the diffusion kinetic equation in particles, which was controlled by both the diffusion rate and the adsorption reaction rate. Above all, hydroxyapatite could efficiently adsorb Fe(III) and prevent the recycling of the iron ion to produce AMD at a low pH value. With the dissolution of HAP, the hydrolysis and precipitation of Fe(III) play important roles in the transformation process. Due to the wide sources and cleanness of HAP, the migration and transformation of Fe(III) in the presence of HAP reported in this study provide an efficiency method for the source control of AMD.

## Figures and Tables

**Table 1:** Element distribution and the Ca to P ratio in the presence of HAP.

Element	HAP	The Reaction Solid Production at pH 2	The Reaction Solid Production at pH 5
Atomic/% of Ca	21.23	10.62	16.65
Atomic/% of P	16.40	15.10	14.26
Atomic/% of O	62.37	72.52	68.76
Atomic/% of Fe	0	1.77	0.33
Ca/P	1.29	0.70	1.17

**Table 2:** Correlations coefficients and sorption parameters obtained using Langmuir, Sips and Freundlich models.

Model	Equation	Sorption Parameters	R^2^
Langmuir	$\frac{C_{t}}{q}=\frac{C_{t}}{q_{m}}+\frac{1}{q_{m}K_{L}}$	*q_m_* = 2.810 (mmol/g) *K_L_* = 19.403 (L/mmol)	0.999
Freundlich	*q* = *K*·*C_t_*^n^	*K* = 2.492 (mmol^n−1^L^n^g^−1^) *n* = 0.232	0.942
Sips	$q=\frac{q_{m}K_{s}C_{t}^{N_{s}}}{1+K_{s}C_{t}^{N_{s}}}$	*q_m_* = 2.81 (mmol/L) *k_S_* = 19.40 (L/mg) *N_S_* = 1	0.993

**Table 3:** Thermodynamic parameters of Fe(III) on HAP.

ΔG^0^/kJ/mol			ΔH^0^ (kJ/mol)	ΔS^0^ (J/(mol·K))
293 K −24.051	313 K	333 K		
	−26.189	−28.588	9.197	113.4

**Table 4:** Adsorption kinetic parameters of Fe(III) onto HAP.

Methods	Equation	Parameters	R^2^
Pseudo-first-order	$\log(q_{m}-q)=\log q_{m}-\frac{k_{ad}t}{2.303}$	*k_ad_* = 0.0056 (min^−1^)	0.807
		*q_m_* = 253.42 (mg/g)	
Pseudo-second-order	$\frac{t}{q}=\frac{1}{k_{ps}q_{m}{}^{2}}+\frac{t}{q_{m}}$	*k* = 0.0011(g/mg·min)	0.996
		*q_m_* = 160.04 (mg/g)	
Particle diffusion	$q=k_{p}t^{1/2}+C$	*k_p_* = 6.774(min^−1^)	0.895
		*C* = 6.774	

## Data Availability

Not applicable.
